# Psychological Distress, Disorder Severity, and Perception of Positive Contributions in Couples Raising Individuals With Autism

**DOI:** 10.3389/fpsyg.2021.694064

**Published:** 2021-06-29

**Authors:** Cristina García-López, Patricia Recio, Pilar Pozo, Encarnación Sarriá

**Affiliations:** ^1^Joint Research Institute National University for Distance Education and Health Institute Carlos III (IMIENS), Madrid, Spain; ^2^Neurology Department, School Learning Disorders Unit (UTAE), Hospital Sant Joan de Déu, Barcelona, Spain; ^3^Department of Methodology of Behavioral Sciences, Faculty of Psychology, National University for Distance Education (UNED), Madrid, Spain

**Keywords:** autism spectrum disorder, positive contributions, severity of autism, actor-partner interdependence model, psychological distress, parents

## Abstract

Parents' perception of the positive contributions associated with raising children with autism is considered to be a protective factor in the process of psychological adaptation. Thus, it is essential to unveil what factors are related to this perception. We explore how parents' psychological distress (parental stress and anxiety) predicts the perception of positive contributions in fathers and mothers who raise individuals with different levels of autism severity. The sample comprises 135 couples (270 fathers and mothers) parenting individuals diagnosed with autism aged 3–38 years. Participants completed different self-report questionnaires, including measures of parental stress, anxiety, and positive contributions. To estimate the actor–partner interdependence model, data were analyzed using structural equation modeling (SEM) to explore transactional effects between fathers' and mothers' psychological distress and their perceptions of positive contributions associated with autism. Two separate multigroup models were tested, respectively, analyzing parental stress and anxiety. Each multigroup model considers two levels of disorder severity. The findings revealed that actor and partner effects of stress and anxiety were important predictors of the perception of positive contributions in both disorder severity groups. We conclude that it is necessary to develop family support programs that focus on controlling fathers' and mothers' stress and anxiety symptoms, as these mental states negatively impact the ability to perceive positive contributions.

## Introduction

Autism spectrum disorder (ASD) is a neurodevelopmental disorder (DSM-5; American Psychiatric Association, 2013) with an especially great impact on the family system. Research has traditionally emphasized the negative outcomes linked to parenting a child with autism, framing the experience as stressful. However, negative and positive aspects coexist when educating a child with autism (Mullins, 1987; Hastings and Taunt, 2002), and the positive aspects have rapidly become apparent since researchers considered them in recent years (Sarriá and Pozo, 2015).

Among the available literature on families raising individuals with autism, there is evidence that some parents not only overcome their child receiving an ASD diagnosis but also grow stronger and experience a new lease of life (Bayat, 2007). Some of the most gratifying facets of educating an individual with autism include fulfillment when caring for the child and helping them develop, a stronger couple and family union, new aims in life, the development of new competences, and individual growth (Taunt and Hastings, 2002; Waizbard-Bartov et al., 2019). However, despite the recent emergence of positive contributions as a relevant research topic (Meleady et al., 2020), very little is known about what factors explain differences in perception of positive contributions among parents.

In the 1980s, Turnbull and colleagues began studies centered on the positive contributions of raising children with disabilities (Turnbull et al., 1986; Summers et al., 1989). Later, Behr (1990) conducted a seminal study on the positive contributions of children with disabilities to the family, which led to the elaboration of the Kansas Inventory of Parental Perceptions (KIPP; Behr et al., 1992).

The inventory's Positive Contribution Scale (PCS) defines nine dimensions of positive contributions: (a) learning through experience with special problems in life; (b) happiness and fulfillment; (c) personal strength and family closeness; (d) understanding life's purposes; (e) personal growth and maturity; (f) awareness of future issues; (g) expanded social network; (h) career or job growth; and (i) pride and cooperation. In a subsequent study that factor-analyzed the PCS (Hastings et al., 2002), these dimensions were grouped into three distinct factors that have become a benchmark in subsequent research: (1) happiness and fulfillment, (2) strength and family closeness, and (3) personal growth and maturity.

In the context of parental adaptation to ASD, the concept of positive contributions represents parents' positive perceptions and feelings toward the child with autism. This idea has been conceptualized through different terms (Meleady et al., 2020), such as positive gain (Jones et al., 2014); positive appraisal (King et al., 2009; Stuart and McGrew, 2009; Paynter et al., 2013; McGrew and Keyes, 2014; Xue et al., 2014; Schlebusch and Dada, 2018); benefit finding (Pakenham et al., 2004; Samios et al., 2009, 2012; Ekas et al., 2015, 2016; Lim and Chong, 2017); positive perceptions (Hastings et al., 2005; Griffith et al., 2010; Ewles et al., 2014; Wong et al., 2016); and positive contributions (Kayfitz et al., 2010; Sarriá and Pozo, 2015; García-López et al., 2016a).

As for gender differences, mothers seem to hold more positive perceptions than fathers of their children with autism (Hastings et al., 2005; Samios et al., 2009; Kayfitz et al., 2010; Pozo et al., 2011; Ekas et al., 2015), although other studies have not found significant differences between mothers and fathers (Xue et al., 2014).

Studies have found various associations between parents' perception of positive contributions regarding ASD and their psychological adaptation. For instance, a negative relationship was reported between maternal perception of positive contributions and anxiety (Pozo et al., 2011; Ekas et al., 2019), while a negative association has been found between positive perceptions and psychological distress in both fathers and mothers (Kayfitz et al., 2010; Wong et al., 2016). In the same line, a negative appraisal of autism by parents positively correlated with individual, marital, and family burden (Stuart and McGrew, 2009). Predictive models of psychological adaptation have shown that positive contributions or benefit finding predict anxiety (Samios et al., 2012), parental stress and anxiety (García-López et al., 2016a), depression and anxiety (Lovell and Wetherell, 2020), and psychological wellbeing (Sarriá and Pozo, 2015).

Considering the relevance of perceived positive contributions to psychological adaptation in parents of individuals with autism, it is essential to unveil what factors predict this perception. However, there is a lack of studies examining the predictors of perceived positive contributions. Hastings et al. (2005) explored both child and partner variables as predictors of parents' perception of positive contributions using 41 mother–father dyads. Their systemic analysis identified mother's mental health (depression) as a significant negative predictor of father's positive perceptions, while neither children's behavior problems nor father's mental health (anxiety or depression) predicted mother's positive perceptions. More studies adopting a systemic perspective are needed to gain better knowledge of which variables predict perceived positive contributions.

Symptom severity has been identified as a risk factor in caring for persons with autism (Benson and Karlof, 2009; Ekas and Whitman, 2010). The frequency with which children manifest symptoms linked to an ASD also contributes to parents' negative psychological outcomes such as stress (Bravo, 2005; Konstantareas and Papageorgiou, 2006; Pozo and Sarriá, 2014; Nieto et al., 2017) and anxiety (Firth and Dryer, 2013; Chan and Leung, 2021). In fact, in most family adaptation models (McCubbin and Patterson, 1983; Patterson, 1988; Seligman and Darling, 2007), child characteristics including disorder severity have been positively associated with heightened stress and anxiety levels among parents (Bebko et al., 1987; Konstantareas and Homatidis, 1989; Bravo, 2005; Chan et al., 2018).

Furthermore, in their systematic review on perceived positive contributions among parents of children with autism, Meleady et al. (2020) recommend further investigation of confounding factors, such as symptom severity and behavior problems, and how these impact on parents' perception of positive contributions. Findings suggest that parental outcomes may be more attributable to ASD-associated challenges, rather than to the diagnosis alone. In particular, behavior problems have repeatedly been demonstrated as the strongest predictor of parental wellbeing among child-related characteristics (Manning et al., 2011; Karst and Van Hecke, 2012; Yorke et al., 2018; Blacher and Baker, 2019). By contrast, findings on the impact of disorder severity on parental outcomes are less consistent.

Taken together, the perception of positive contributions of raising a child with autism is increasingly demonstrating its relevance to promoting positive adaptation; consequently, its inclusion in family adaptation studies is amply justified. However, scarce research has explored what variables predict perceived positive contributions in families raising individuals with different severity levels of ASD. In addition, most studies in this field have only considered the adaptation of mothers, with very few focusing on both parents. Likewise, most statistical analyses have not considered the interactions occurring within the couple, affecting each parent at a cognitive, emotional, and behavioral level. This interdependence implies the need to consider a systemic perspective when studying family adaptation in the context of ASD (García-López et al., 2016a,b,c).

Considering the limitations identified in previous research, this study explores the notion that parental positive perceptions might be related not only to one's own psychological distress but also to that of one's partner. In particular, we adopt a transactional perspective to analyze the relationship between parental distress (parental stress and anxiety) and the perception of positive contributions associated with raising an individual with autism. Specifically, we use structural equation modeling (SEM) to estimate the actor–partner interdependence model (APIM; Kenny, 1996; Kenny and Cook, 1999). Two positive contributions are studied in our model: source of strength and family closeness and source of happiness and fulfillment. Due to the potential importance of disorder severity in the relationships of these variables, we used a multigroup model with two levels of severity.

## Methods

### Participants

The sample includes 135 biological Spanish father–mother dyads raising individuals with autism. None of the families had any other children with disabilities. Participants were aged 28–72 years, with no significant difference between the ages of mothers (*M* = 43.23; *SD* = 6.75) and fathers (*M* = 44.88; *SD* = 7.62). However, we found a significant difference in employment (χ^2^ = 51.61, *p* < 0.001): 79.3% of fathers were employed full-time, compared to 48.1% of mothers. Parents' sociodemographic characteristics and family income are reported in Table 1. The inclusion criteria required having a son or daughter with the diagnosis of ASD and both parents living in the same house. A qualified psychologist made the diagnosis according to the *Diagnostic and Statistical Manual of Mental Disorders, 4*^*th*^ *Edition, Text Revision* (DSM-IV-TR; American Psychiatric Association, 2000) or *5*^*th*^ *Edition* (DSM-5; American Psychiatric Association, 2013), according to the existing criteria at the time of diagnosis.

**Table 1:** Parents' sociodemographic characteristics and family income.

**Characteristic**	**Fathers**	**Mothers**
	**% (*n*)**	**% (*n*)**
**Education level**		
Primary school	12.6 (17)	12.6 (17)
Secondary school	42.2 (57)	36.3 (49)
University	45.2 (61)	51.2 (69)
**Employment**		
Full-time^a^	79.3 (107)	48.1 (65)
Part-time^b^	2.2 (3)	7.4 (10)
Unemployed	4.4 (6)	9.6 (13)
Other	14.1 (19)	34.8 (47)
**Family income^c^** **(**€**)**	**Family**	
<500	23.0 (62)	
500–850	30.4 (82)	
850–1,200	18.5 (50)	
1,200–1,800	16.3 (44)	
**Number of children**		
One	36 (49)	
Two	54.4 (74)	
Three or more	9.6 (13)	

a *Full-time: 40 h per week*.

b *Part-time: 20 h per week*.

c *Family income: monthly income per family member*.

The age range of individuals with autism was 3–38 years (*M* = 11.14; *SD* = 6.65); most attended an ordinary school; 83.8% were male and 16.2% were female. The most frequent diagnosis was autistic disorder, followed by ASD. Table 2 reports the descriptive statistics of individuals with autism. Because we aimed to analyze the role of disorder severity in the relationship between psychological distress and perceived positive contributions, we divided the sample into two groups: one included families raising individuals with mild-to-moderate ASD (*n* = 89) and the other included families of individuals with severe ASD (*n* = 46).

**Table 2:** Descriptive characteristics of individuals with autism.

**Characteristic**	**% (*n*)**
**Diagnosis type**	
ASD (DSM-V criteria)	24.3 (33)
Autistic disorder	39.7 (54)
Asperger syndrome	8.8 (12)
PDD-NOS^a^	14.7 (20)
Rett syndrome	5.9 (8)
Childhood disintegrative disorder	14.7 (20)
**Education center type**	
Ordinary school	57.4 (78)
Special education school	8.8 (12)
Autism-specific school	27.2 (37)
Day center	3.7 (5)
Others	2.9 (4)
**Disorder severity**	
Mild-moderate	65.9 (89)
Severe	34.1 (46)

a *Pervasive developmental disorder—not otherwise specified*.

Participants were recruited from the Learning Disabilities Unit (UTAE) at Hospital Sant Joan de Déu in Barcelona, education centers in Madrid (ALEPH, CEPRI, PAUTA, Antonio Gala, and Enrique Tierno Galván), parents' associations (PROTGD, Autismo Burgos), and the Spanish Professional Association of Autism (AETAPI). The parents were given a letter that described the purpose and procedures of the study. Participation was voluntary and involved filling in different questionnaires on paper or online. As compensation for their time, participants had the chance to receive the results of their personal psychological adaptation profiles after completing the questionnaires.

### Instruments

Sociodemographic variables were collected through a questionnaire specially constructed for this investigation. All other instruments used in this study had been previously adapted into Spanish by other authors.

#### Childhood Autism Rating Scale

The CARS was originally devised by Schopler et al. (1988), then adapted to Spanish by García-Villamisar and Polaino-Lorente (1992). This scale comprises 15 items that clinicians score from 1 (*age-appropriate behavior*) to 4 (*severe or profoundly abnormal behavior*). Total scores range from 15–60; scores higher than 30 indicate the presence of ASD. The posterior study by Chlebowski et al. (2010) confirmed the utility of the CARS in differentiating ASD from other developmental disorders and typical development and suggests that treating a score of 25 as the cut-off for ASD, which is common in clinical practice, is a valid approach. Accordingly, we considered scores from 25 to 36.5 to indicate mild-to-moderate ASD and scores of 37 or higher to indicate severe ASD. In the present study, the psychologists who treated the children of participants applied this scale. The CARS showed good internal consistency in our sample (α = 0.93).

#### Parental Stress Index

The PSI was originally formulated by Abidin (1995). To measure parental stress levels, we used the Spanish version of the PSI Short Form (PSI/SF; Díaz-Herrero et al., 2011), a self-administered scale of 36 items scored on a 5-point Likert scale from 1 (*completely disagree*) to 5 (*completely agree*). The internal consistency of the global scale was 0.91. The PSI/SF contains three subscales: (a) parental distress, (b) dysfunctional parent–child interaction, and (c) a difficult child. As demonstrated by Zaidman-Zait et al. (2010), items in the parental distress subscale are useful to assess the severity of distress among parents of individuals with autism. By contrast, items in the dysfunctional parent–child interaction and difficult child subscales were not developed considering the specific behavioral profile of children with autism, and thus function less well. Therefore, following Zaidman-Zait et al.'s (2010) recommendation, we used the parental distress subscale, which showed good internal consistency in our sample (α = 0.87).

#### Hospital Anxiety and Depression Scale

The HADS was devised by Zigmond and Snaith (1983), then adapted and validated in a Spanish sample by Tejero et al. (1986). This 14-item self-administered questionnaire includes two subscales—for anxiety and depression—with responses to each item given on a 4-point Likert scale ranging from 0 to 3. We used the anxiety subscale (HADS-A). Total scores of 12 or higher indicate clinically significant levels of anxiety. In a literature review of the validity of the HADS (Bjelland et al., 2002), the Cronbach's α of HADS-A varied from 0.68 to 0.93 (*M* = 0.83). The HADS-A subscale showed good internal consistency in our sample (α = 0.90).

#### Positive Contribution Scale of the Kansas Inventory of Parental Perceptions

Behr et al. (1992) devised the KIPP, which is available in Spanish on the Beach Center website. This scale comprises 50 self-reported items evaluating the positive feelings of parents toward their child with autism, organized in nine subscales. For each item, scores range from 1 (*strongly disagree*) to 4 (*strongly agree*). Higher scores are associated with more positive perceptions. We calculated two subscales: (a) source of happiness and fulfillment, to assess positive feelings toward the child, and (b) source of strength and family closeness, to represent the positive impact on the family. These subscales have shown good reliability in previous studies using Spanish populations (García-López et al., 2016a). In our study, the Cronbach's α for the source of happiness and fulfillment and source of strength and family closeness subscales were 0.78 and 0.83, respectively.

### Data Analysis

Relevant variables for fathers and mothers were compared using ANOVA. Pearson's correlation coefficients were used to determine the relationships among the study variables. Before using the maximum-likelihood estimation procedure in SEM, we first checked that the normality assumption was not severely violated (Curran et al., 1996). According to the thresholds of severe non-normality (i.e., skewness > 3; kurtosis > 10) proposed by Kline (2005), the studied variables can be regarded as fairly normally distributed, and thus suitable for further analyses.

**Figure 1:**
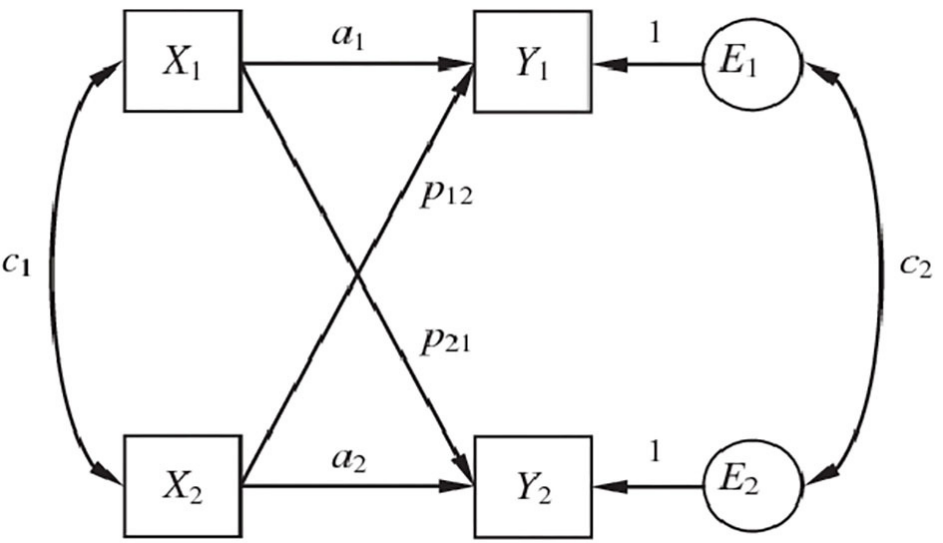
Theoretical APIM model.

To investigate the effects of parental stress and anxiety on the perception of positive contributions, data were analyzed using SEM to estimate the actor–partner interdependence model (APIM; Kenny, 1996; Kenny and Cook, 1999). The APIM allows the study of transactional effects between fathers and mothers within the same family unit, and accounts for the interdependence of the observations. Two types of effects can be calculated using the APIM: actor and partner effects. Actor effects estimate the extent to which a predictor variable of one parent is related to his/her own outcome variable (in our study, a perceived positive contribution associated with raising an individual with autism); partner effects measure the degree to which a predictor variable of one parent is related to his/her partner's outcome variable. The model includes correlations between the two independent variables and correlations between the two residual variables. The correlation between the independent variables ensures that actor effects are estimated controlling for partner effects, and vice versa. The correlation between error terms considers that the unexplained variance in the outcome variables is correlated, even after removing the covariance due to partner effects, thus controlling for additional sources of non-independence (see Figure 1). The data were analyzed using AMOS 21 (Arbuckle, 2006).

We tested two separate multigroup models for parental stress and anxiety, with each multigroup model considering two levels of disorder severity. To evaluate model fit, we used several different fit indices: chi-square statistic, the ratio of chi-square to its degree of freedom (<3 indicating acceptable fit), root mean square error of approximation (RMSEA), comparative fit index (CFI), and normed fit index (NFI). Root mean square error of approximation criteria were as follows: <0.05, good fit; 0.05–0.08, satisfactory fit; 0.08–0.10, mediocre fit; >0.10, unacceptable fit. Values of CFI and NFI range between 0 and 1: 0.90–0.95 indicates acceptable model fit; >0.95 indicates good model fit (Browne and Cudeck, 1992).

Finally, the chi-square difference (likelihood ratio) statistic (Δχ^2^) was used to compare the fit for two nested models. Significant values on the chi-square difference test indicate that the constraints on the more restricted model may be too strict and that the results of the less restricted model should be accepted, following the recommendation by Cheung and Rensvold (2002).

## Results

### Preliminary Analysis

As preliminary analysis, we conducted descriptive statistics (Table 3) and ANOVA for the studied variables. Four mixed ANOVAs were applied to compare the mean differences in the studied variables (parental stress, anxiety, strength/family closeness, and happiness/fulfillment), with mothers/fathers as the related-measures factor and disorder severity as the between-subjects factor. There were no significant differences in anxiety and parental stress between mothers and fathers or between levels of disorder severity, but there were statistically significant main effects for both factors in the measures of positive contributions.

**Table 3:** Descriptive statistics of the studied variables in mothers and fathers of the two ASD severity groups.

**Variable**	**Fathers (*****n*** **=** **135)**		**Mothers (*****n*** **=** **135)**	
	**MM ASD**	**Severe ASD**	**MM ASD**	**Severe ASD**
	**Mean (*SD*)**	**Mean (*SD*)**	**Mean (*SD*)**	**Mean (*SD*)**
Parental stress	30.00 (9.27)	35.65 (8.09)	31.30 (9.09)	35.61 (8.32)
Anxiety	7.03 (3.88)	7.52 (3.60)	7.47 (3.81)	8.67 (3.60)
Strength/family closeness	20.60 (3.31)	18.72 (3.18)	23.97 (2.92)	22.37 (2.81)
Happiness/fulfillment	18.85 (2.98)	16.50 (3.18)	20.11 (3.36)	17.61 (3.82)

*MM, Mild-moderate*.

The results showed statistically significant differences between mothers and fathers in both measures of perceived positive contributions: strength/family closeness [*F*_(1, 133)_ = 161.31, *p* < 0.001, η^2^ = 0.55] and happiness/fulfillment [*F*_(1, 133)_ = 16.82, *p* < 0.001, η^2^ = 0.11]. Compared to fathers, mothers presented a significantly higher perception of strength/family closeness (Mothers: *M* = 23.17, *SD* = 2.97; Fathers: *M* = 19.71, *SD* = 3.38) and happiness/fulfillment (Mothers: *M* = 18.86, *SD* = 3.80; Fathers: *M* = 17.67, *SD* = 3.29).

In relation to disorder severity, ANOVA also revealed significant differences between groups in both measures of perceived positive contributions: strength/family closeness [*F*_(1, 133)_ = 12.78, *p* < 0.001, η^2^ = 0.09) and happiness/fulfillment [*F*_(1, 133)_ = 21.48, *p* < 0.001, η^2^ = 0.14]. Parents of children with severe ASD scored significantly lower in perceived strength/family closeness (*M* = 20.54, *SD* = 3.50) and perceived happiness/fulfillment (*M* = 17.05, *SD* = 3.54) compared with parents of children with mild-moderate ASD (*M* = 22.28, *SD* = 3.54; *M* = 19.48, *SD* = 3.23, respectively). The interaction effects between gender and disorder severity were not significant.

We assessed the associations between parental stress, anxiety, and perceived positive contributions (happiness/fulfillment and strength/family closeness) through bivariate correlations. Table 4 shows the correlations between the studied variables separately for mothers and fathers. All correlation coefficients are in the same direction and of roughly similar sizes for mothers and fathers, except for the relationships between anxiety and the two subscales of perceived positive contributions. Specifically, the relationships of anxiety with strength/family closeness and happiness/fulfillment were significant for mothers (−0.22, −0.29) but not for fathers (−0.16, −0.14). There were significant inter-correlations between the measures of perceived positive contributions and between parental stress and anxiety. Table 5 shows the correlations between mothers' and fathers' scores for the studied variables. The diagonal of this table reveals statistically significant correlations between fathers' and mothers' parental stress, anxiety, perceived strength/family closeness, and perceived happiness/fulfillment. These correlations indicate that couple members' scores are not independent, which confirms the value of dyad-level analyses (Kenny et al., 2006).

**Table 4:** Pearson correlations between studied variables in mothers and fathers.

	**1**	**2**	**3**	**4**
1. Parental stress	–	0.61^**^	−0.23^**^	−0.29^**^
2. Anxiety	0.64^**^	–	−0.22^**^	−0.25^**^
3. Strength/family closeness	−0.29^**^	−0.16	–	0.54^**^
4. Happiness/fulfillment	−0.29^**^	−0.14	0.52^**^	–

*Values above the diagonal are for mothers and those below the diagonal are for fathers*.

** *p < 0.01*.

**Table 5:** Pearson correlations between mothers' and fathers' scores for studied variables.

**Variables**	**1 (M)**	**2 (M)**	**3 (M)**	**4 (M)**
1. Parental stress (F)	0.37^**^	0.29^**^	−0.29^**^	−0.16
2. Anxiety (F)	0.28^**^	0.27^**^	−0.17^*^	−0.02
3. Strength/family closeness (F)	−0.16	−0.20^*^	0.54^**^	0.41^**^
4. Happiness/fulfillment (F)	−0.16	−0.26^**^	0.40^**^	0.59^**^

*F, Father; M, Mother*.

* *p < 0.05*;

** *p < 0.01*.

### SEM Analysis

We then tested the two models. Both the parental stress model [χ^2^_(3)_ = 1.32, *p* = 0.724; χ^2^*/d.f*. = 0.44; CFI = 1.00; NFI = 0.990; RMSEA = 0.000] and the anxiety model [χ^2^_(1)_ = 0.42, *p* = 0.516; χ^2^*/d.f*. = 0.42; CFI = 1.00; NFI = 0.998; RMSEA = 0.000] showed good fit. These models were modified to exclude some non-significant path coefficients, taking into account the information provided by the modification indices. The goodness-of-fit test for single-group analysis cannot detect possible differences across groups of parameters in the model. To analyze these differences, we require the model specifications to incorporate parameters specific to each level of disorder severity. Accordingly, we tested a multigroup model for the two groups. One requirement for multigroup model analysis is that the model presents a good fit for each of the groups separately. This preliminary analysis presented indicators of good fit in the four models tested: parental stress model of the mild-moderate ASD group (χ^2^ = 0.243, *p* = 0.970; CFI = 1.00; NFI = 0.998; RMSEA = 0.000); parental stress model of the severe ASD group (χ^2^ = 1.954, *p* = 0.582; CFI = 1.00; NFI = 0.975; RMSEA = 0.000); anxiety model of the mild-moderate ASD group (χ^2^ = 0.24, *p* = 0.876; CFI = 1.00; NFI = 1.00; RMSEA = 0.000); anxiety model of the severe ASD group (χ^2^ = 1.253, *p* = 0.262; CFI = 0.996; NFI = 0.985; RMSEA = 0.075).

**Figure 2:**
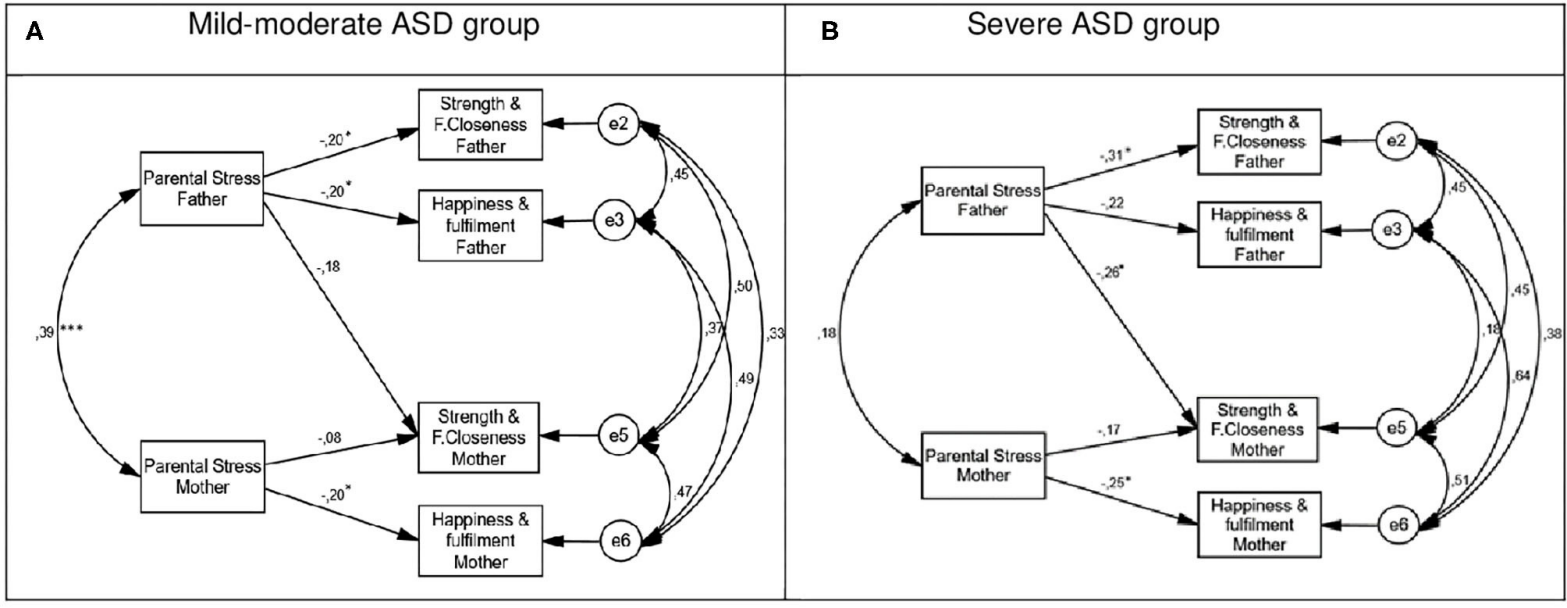
Multigroup parental stress model. **(A)** Mild moderate ASD group. **(B)** Severe ASD group. *p < 0.05; ***p < 0.001.

**Figure 3:**
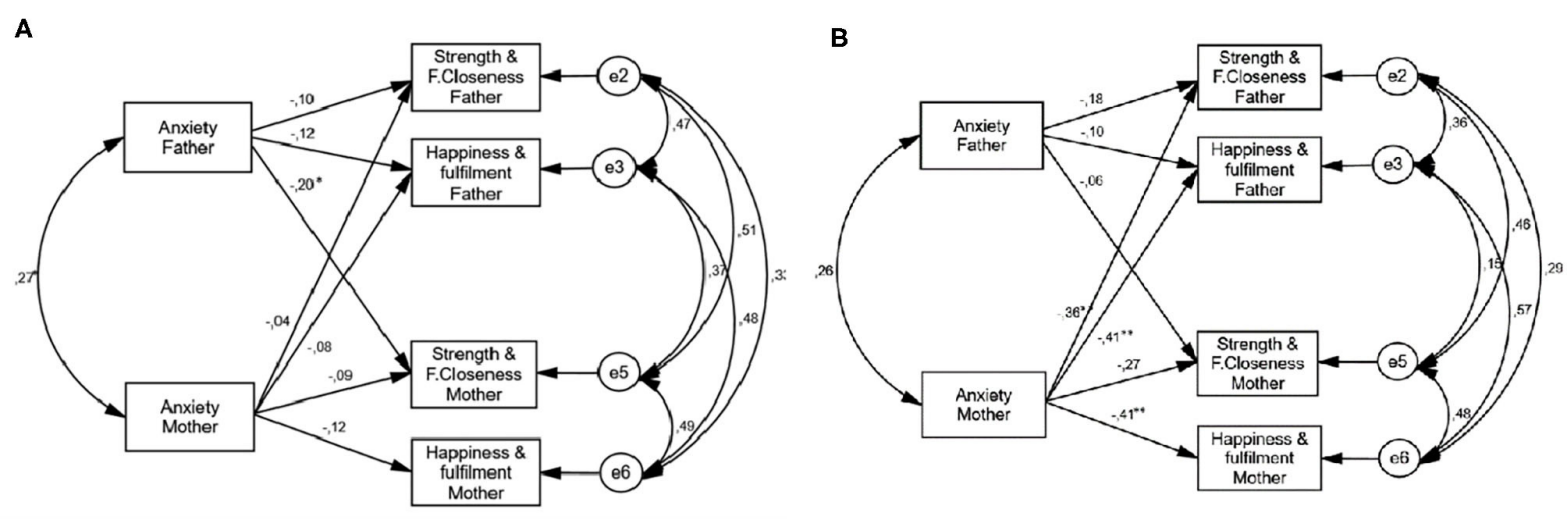
Multigroup parental anxiety model. **(A)** Mild moderate ASD group. **(B)** Severe ASD group. *p < 0.05; **p < 0.01.

Before testing for invariance, we tested the model fit for the samples of mild-moderate and severe ASD. The results indicated a good fit of the model to the data. We then performed various tests of invariance. Paths, error variances, and correlations were allowed to vary across groups (baseline model). This completely unconstrained model showed a very good fit in both the parental stress (χ^2^ = 2.21, *p* = 0.890; CFI = 1.00; NFI = 0.988; RMSEA = 0.000) and anxiety models (χ^2^ = 1.29, *p* = 0.526; CFI = 1.00; NFI = 0.993; RMSEA = 0.000). These values indicate that configural invariance is attained and that the pattern of fixed and non-fixed parameters in the research model is identical for the mild-moderate and severe ASD samples. The parameter estimates for both samples are shown in Figures 2, 3.

We then compared a model in which all paths were allowed to vary across groups with a model in which all paths were set to be equal across groups. The results indicated that the less constrained model did not fit the data significantly better than the fully constrained model (Δχ^2^ = 97.76; Δ*df* = 39, *p* < 0.010; ΔCFI = 0.054) for parental stress (Δχ^2^ = 1.36; Δ*df* = 5, *p* = 0.930) or anxiety (Δχ^2^ = 8.47; Δ*df* = 7, *p* = 0.290). Therefore, we can conclude that the path coefficients do not differ for mild-moderate and severe ASD. We drew the same conclusion for the structural covariances model and the structural residuals model. For each model we report the actor effects for fathers and mothers, the partner effects running from fathers to mothers, and the partner effects running from mothers to fathers (see Figures 2, 3).

#### Actor and Partner Effects

##### Mild-Moderate ASD Group

In the parental stress model of the mild-moderate ASD group, actor effects were found in the relationship between parental stress and their own perceived happiness/fulfillment for both mothers (β = −0.20, *p* < 0.05) and fathers (β = −0.20, *p* < 0.05). An extra actor effect was found for fathers, with parental stress negatively associated with perceived strength/family closeness (β = −0.20, *p* < 0.05; see Figure 2). In the anxiety model, no actor effects were found (see Figure 3). Only one partner effect was found in the mild-moderate ASD group: father's anxiety negatively predicted mother's perception of strength/family closeness (β = −0.20, *p* < 0.05).

##### Severe ASD Group

In the parental stress model of the severe ASD group, two actor effects were found: in the relationship between parental stress and perceived happiness/fulfillment for mothers (β = −0.25, *p* < 0.05) and in the relationship between parental stress and perceived strength/family closeness for fathers (β = −0.31, *p* < 0.05). In the anxiety model there was one actor effect: a negative association between anxiety and perceived happiness/fulfillment for mothers (β = −0.41, *p* < 0.01). There were three partner effects in the severe ASD group: one in the parental stress model and two in the anxiety model (see Figures 2, 3). Father's stress was negatively associated with mother's perceived strength/family closeness, while mother's anxiety was negatively associated with father's perceived strength/family closeness (β = −0.36, *p* < 0.01) and perceived happiness/fulfillment (β = −0.41, *p* < 0.01).

## Discussion

Parenting an individual with autism entails families experiencing increased levels of stress and anxiety (Hastings and Brown, 2002; Little, 2002; Hastings, 2003; Hastings et al., 2005; Lee, 2009; Dabrowska and Pisula, 2010; Gau et al., 2012; Chan and Leung, 2021). Prior research has identified various risk and protective factors during the process of psychological adaptation. Among the recognized protective factors is parents' perception of positive contributions of raising a child with autism (Samios et al., 2012; Sarriá and Pozo, 2015; García-López et al., 2016a; Lovell and Wetherell, 2020), although knowledge of what factors explain the variability in this perception remains scarce. By contrast, symptom severity has been identified as a risk factor in caring for individuals with autism (Benson and Karlof, 2009; Ekas and Whitman, 2010), with studies suggesting that negative parental outcomes may be more attributable to ASD-associated challenges, rather than to the diagnosis alone. Specifically, the presence of intense behavior problems and greater disorder severity have been associated with negative parental outcomes. While behavior problems have repeatedly been found to strongly predict parental wellbeing (Manning et al., 2011; Karst and Van Hecke, 2012; Blacher and Baker, 2019), there is less consistency in findings on the impact of disorder severity.

This study analyzed how a parent's psychological distress (parental stress and anxiety) predicts their own and their partner's perception of positive contributions in raising individuals with different levels of ASD severity. Parents of children with severe ASD reported a significantly lower perception of positive contributions compared with parents of children with mild-moderate ASD. Although fathers and mothers did not show differences in parental stress and anxiety, consistent with earlier findings (Hastings et al., 2005; Samios et al., 2009; Kayfitz et al., 2010; Pozo et al., 2011; Ekas et al., 2015), mothers perceived higher levels of positive contributions than did fathers. Most studies in this field have only considered mothers' adaptation, with very few focusing on both parents and offering insights on gender differences. Future studies of adaptation in the context of ASD should adopt a systemic perspective, including mothers and fathers as well as potential explanatory variables, such as care-giving involvement, that might explain gender differences (García-López et al., 2016a,b,c). In the correlational analysis, most correlation coefficients were in the same direction and of roughly similar sizes for mothers and fathers. The exceptions were the negative relationships between anxiety and the two subscales of perceived positive contributions, which were only significant in mothers. There were significant associations between mothers' and fathers' levels of psychological distress and positive contributions, which justified applying the APIM.

As shown by the multigroup SEM analysis, in the mild-moderate ASD group there were three actor effects in the parental stress model and one partner effect in the anxiety model. Specifically, father's stress was negatively related to perceived strength and family closeness and perceived happiness and fulfillment (actor effects); mother's stress was negatively related to perceived happiness and fulfillment (actor effect); and father's anxiety was negatively related to mother's perceived strength and family closeness (partner effect). These results emphasize that for parents raising an individual with mild-moderate ASD, the father's psychological distress is related to both partners' ability to perceive positive contributions. Once again, these results reinforce the need to analyze samples equally representing fathers and mothers and examine the transactional patterns of influence between partners. Although the multigroup SEM analysis did not allow us to conclude that path coefficients differ for mild-moderate and severe ASD, a comparison between the two groups reveals that both had three actor effects but the severe ASD group had more partner effects (three) than the mild-moderate ASD group (one). These results suggest that disorder severity might strengthen the presence of partner effects, especially for mothers. In particular, when the child's ASD is severe, mother's anxiety is negatively related to both parents' ability to perceive positive contributions.

Other gender differences should be noted. For mothers, anxiety has a greater impact than parental stress on both their own perception of positive contributions and that of their partner; for fathers, by contrast, parental stress has a greater impact than anxiety on both their own and their partner's ability to perceive positive contributions. These results are in line with previous research in this area. Specifically, Pozo et al. (2011) found a negative relationship between maternal positive contributions and anxiety, while Kayfitz et al. (2010) reported a negative association between positive perceptions and stress in both fathers and mothers.

These results reinforce the previously identified need to raise awareness of the transactional effects between the parenting and couple domains. In particular, Goetz et al. (2019) found that a negative perception of the interactions with one's partner, caused by that partner's stress, could reduce the likelihood of perceiving the positive contributions that a child with autism can make to the family. Most statistical analyses have not considered within–couple interactions. We should, therefore, focus especially on strengthening parents' positive cognitions in difficult times and promoting the positive impact of these cognitions on the couple and the whole family system through transactional effects.

Another important aspect is that family resilience alleviates the association between maternal psychological distress and the severity of children's developmental disorders (Suzuki et al., 2018). Resilience processes include, for example, making positive meaning of a disability, mobilizing resources, and coming closer as a family (Bayat, 2007). Accordingly, future studies should analyze the potential role of family resilience in promoting more positive perceptions in parents.

This research has several limitations that need to be considered. First, our sample only included heterosexual couples able to access clinical services. Therefore, future studies should include other family structures and aim to achieve larger samples recruited by diverse means. Second, although SEM is an advanced statistical approach for analyzing dyadic data, causal inferences cannot be made. Third, reliance on some self-report instruments and measurements reduces measurement reliability. In particular, while the PSI-SF is widely used for measuring parental stress in families of children with autism, studies examining its psychometric properties have questioned its factorial structure (Reitman et al., 2002; Zaidman-Zait et al., 2011) and detected that several items on two subscales (dysfunctional parent–child interaction and difficult child) have poor ability to discriminate variability of parental stress in such families (Zaidman-Zait et al., 2010). Consequently, as recommended by Zaidman-Zait et al., 2010, we used only the score from the parental distress subscale. Fourth, as this is a cross-sectional study, there is a need to carry out longitudinal studies that explore the differential impact of variables associated with positive contributions across different developmental periods, as Meleady et al. (2020) suggested. In addition, we did not collect data on how long the couples had been in a relationship and the time since diagnosis.

Despite these limitations, our study's results highlight the relevance of psychological distress and perception of positive contributions for parental adaptation. We consider that this study is a relevant contribution to the increasingly expansive literature about autism. The latest advances in the understanding of ASD demonstrate the importance of an interdisciplinary approach (Wadhera and Kakkar, 2020). Both basic research, which promotes a better understanding of the psychological processes the disorder entails (Kakkar, 2020), and applied research, such as diagnosis (Wadhera and Kakkar, 2019) or family intervention (Nordahl-Hansen et al., 2018; Shalev et al., 2020), are essential to achieving a better comprehension of autism.

Theory and research implications include support for using the APIM (Kenny, 1996; Kenny and Cook, 1999) in research on families parenting children with autism. We adjusted this theoretical model to the empirical data collected in our study, which allowed us to explore actor and partner effects between mothers and fathers. Furthermore, our findings support the importance of using statistical methods, such as SEM, that account for interdependence when analyzing dyadic data. Our study was also able to emphasize gender adaptation differences by equivalently representing mothers' and fathers' experiences, which reinforces the case for including both parents in future studies.

Regarding practical implications, our findings support the need to focus not only on the child's functioning (such as disorder severity) but also on family adaptability (such as parents' psychological distress and ability to perceive the positive aspects of raising a child with autism). Given that outcomes in individuals with autism are closely related to the family system and the psychological state of family members (Osborne et al., 2008; Baker et al., 2011; Zaidman-Zait et al., 2014; Reid et al., 2019; Rodriguez et al., 2019), it is essential to take care of the parents' psychological wellbeing through clinical interventions and support groups.

Clinical programs and support groups for parents of individuals with autism might be improved by increasing focus on managing parental stress and anxiety through the life cycle of families raising individuals with autism. Psychoeducation and training on specific strategies to enhance psychological wellbeing could enhance parents' ability to perceive positive contributions in raising an individual with autism. Our study highlights the need to preserve parents' psychological wellbeing, thereby equipping them to appreciate the positive aspects of their parenting experience. Various prior studies have identified the ability to perceive positive contributions as an adaptive coping strategy, associated with superior adaptation outcomes such as better mental health (Wong et al., 2016; Ekas et al., 2019) and higher levels of quality of life (Ferrer et al., 2017).

Considering the results of our study, it might also be appropriate to tailor support groups according to ASD severity, since the associations between psychological distress and perception of positive contributions may vary depending on the severity levels. We recommend that support groups be homogeneous in terms of children's behavioral profiles (e.g., mild-moderate or severe ASD), as parents of children with similar features can more easily share relevant experiences and strategies.

Overall, each parent's perception of positive contributions is related to their own psychological distress and that of their partner for both mild-moderate and severe ASD situations. Consequently, promoting the psychological wellbeing of one parent will have positive effects on both mothers and fathers. This implies the need for intervention strategies in family support programs that encourage dyadic support techniques among parents (García-López et al., 2016c; Brown et al., 2020), so that one parent's strengths and positive reframing can promote their own psychological adaptation and that of their partner. Finally, given the transactional relationships found between mothers' and fathers' psychological distress and their perception of positive contributions, it is crucial to encourage both parents to participate in clinical programs and support groups. Their involvement in these interventions will facilitate individual wellbeing and family functioning.

## Data Availability Statement

The original contributions presented in the study are included in the article/supplementary material, further inquiries can be directed to the corresponding author/s.

## Ethics Statement

The studies involving human participants were reviewed and approved by Ethical Committee of National University for Distance Education (UNED). Written informed consent to participate in this study was provided by the participants' legal guardian/next of kin.

## Author Contributions

CG-L, PR, PP, and ES worked together, made substantial contributions to the conception and design of the study, and agreed to be accountable for all aspects of the work. CG-L and PP were involved in the literature review, participant recruitment, data collection, and data entry. PR and ES were involved in data analysis and interpretation. All authors were involved in writing the manuscript, critically revising it for important intellectual content and gave final approval of the version to be published.

## Conflict of Interest

The authors declare that the research was conducted in the absence of any commercial or financial relationships that could be construed as a potential conflict of interest.

## References

[B1] Abidin R. R. (1995). Parenting Stress Index Manual. Odessa, FL: Psychological Assessment Resources.

[B2] American Psychiatric Association (2000). Diagnostic and Statistical Manual of Mental Disorders 4th Edn. Text Revision (DSM-IV-TR). Washington, DC: American Psychiatric Association.

[B3] American Psychiatric Association (2013). Diagnostic and Statistical Manual of Mental Disorders 5th Edn. (DSM-5). Washington, DC: American Psychiatric Association.

[B4] Arbuckle J. L. (2006). AMOS (Version 7.0). User's Guide. Pennsylvania, PA: SPSS.

[B5] Baker J. K. Seltzer M. M. Greenberg J. S. (2011). Longitudinal effects of adaptability on behavior problems and maternal depression in families of adolescents with autism. J. Fam. Psychol. 25, 601–609. 10.1037/a0024409 21668120 PMC3987806

[B6] Bayat M. (2007). Evidence of resilience in families of children with autism. J. Intellect. Disabil. Res. 51, 702–714. 10.1111/j.1365-2788.2007.00960.x 17845239

[B7] Bebko J. M. Konstantareas M. M. Springer J. (1987). Parent and professional evaluations of family stress associated with characteristics of autism. J. Autism Dev. Disord. 17, 565–576. 10.1007/BF01486971 3680156

[B8] Behr S. K. (1990). The Underlying Dimensions of Positive Contributions that Individuals with Developmental Disabilities Make to Their Families: A Factor Analytic Study. Ann Arbor, MI: University Microfilms, Inc.

[B9] Behr S. K. Murphy D. L. Summers J. A. (1992). User's Manual: Kansas Inventory of Parental Perceptions (KIPP). Laurence, Kansas: Beach Center on Families and Disability.

[B10] Benson P. R. Karlof K. L. (2009). Anger, stress proliferation, and depressed mood among parents of children with ASD: a longitudinal replication. J. Autism Dev. Disord. 39, 350–362. 10.1007/s10803-008-0632-0 18709548

[B11] Bjelland I. Dahl A. A. Haug T. T. Neckelmann D. (2002). The validity of the Hospital Anxiety and Depression Scale: an updated literature review. J. Psychosom. Res. 52, 69–77. 10.1016/S0022-3999(01)00296-3 11832252

[B12] Blacher J. Baker B. L. (2019). Collateral effects of youth disruptive behavior disorders on mothers' psychological distress: adolescents with autism spectrum disorder, intellectual disability, or typical development. J. Autism Dev. Disord. 49, 2810–2821. 10.1007/s10803-017-3347-2 29071563

[B13] Bravo K. (2005). “Severity of autism and parental stress: the mediating role of family environment.” in Dissertation Abstracts International: Section A: The Humanities and Social Sciences, Vol. 66 (Ann Arbor, MI: University Microfilms International), 3.821.

[B14] Brown M. Whiting J. Kahumoku-Fessler E. Witting A. B. Jensen J. (2020). A dyadic model of stress, coping, and marital satisfaction among parents of children with autism. Fam. Relat. 69, 138–150. 10.1111/fare.12375

[B15] Browne M. W. Cudeck R. (1992). Alternative ways of assessing model fit. Sociol. Methods Res. 21, 230–258. 10.1177/0049124192021002005

[B16] Chan K. K. S. Lam C. B. Law N. C. W. Cheung R. Y. M. (2018). From child autistic symptoms to parental affective symptoms: a family process model. Res. Dev. Disabil. 75, 22–31. 10.1016/j.ridd.2018.02.005 29455076

[B17] Chan K. K. S. Leung D. C. K. (2021). Linking child autism to parental depression and anxiety: The mediating roles of enacted and felt stigma. J. Autism Dev. Disord. 51, 527–537. 10.1007/s10803-020-04557-6 32519191

[B18] Cheung G. W. Rensvold R. B. (2002). Evaluation goodness-of-fit indexes for testing measurement invariance. Struct. Equ. Model. 9, 233–255. 10.1207/S15328007SEM0902_5

[B19] Chlebowski C. Green J. A. Barton M. L. Fein D. (2010). Using the childhood autism rating scale to diagnose autism spectrum disorders. J. Autism Dev. Disord. 40, 787–799. 10.1007/s10803-009-0926-x 20054630 PMC3612531

[B20] Curran P. J. West S. G. Finch J. F. (1996). The robustness of test statistics to nonnormality and specification error in confirmatory factor analysis. Psychol. Methods. 1, 16–29. 10.1037/1082-989X.1.1.16

[B21] Dabrowska A. Pisula E. (2010). Parenting stress and coping styles in mothers and fathers of pre-school children with autism and Down syndrome. J. Intellect. Disabil. Res. 54, 266–280. 10.1111/j.1365-2788.2010.01258.x 20146741

[B22] Díaz-Herrero A. López-Pina J. A. Pérez-López J Brito de la Nuez A. G. Martínez-Fuentes M. T. (2011). Validity of the parenting stress index-short form in a sample of Spanish fathers. Span. J. Psychol. 14, 990–997. 10.5209/rev_SJOP.2011.v14.n2.44 22059342

[B23] Ekas N. V. Ghilain C. Pruitt M. Celimli S. Gutierrez A. Alessandri M. (2016). The role of family cohesion in the psychological adjustment of non-Hispanic White and Hispanic mothers of children with autism spectrum disorder. Res. Autism Spectr. Disord. 21, 10–24. 10.1016/j.rasd.2015.09.002

[B24] Ekas N. V. Tidman L. Timmons L. (2019). Religiosity/spirituality and mental health outcomes in mothers of children with autism spectrum disorder: the mediating role of positive thinking. J. Autism Dev. Disord. 49, 4547–4558. 10.1007/s10803-019-04165-z 31414262

[B25] Ekas N. V. Timmons L. Pruitt M. Ghilain C. Alessandri M. (2015). The power of positivity: Predictors of relationship satisfaction for parents of children with autism spectrum disorder. J. Autism Dev. Disord. 45, 1997–2007. 10.1037/0022-3514.93.2.285 25601217

[B26] Ekas N. V. Whitman T. L. (2010). Autism symptom topography and maternal socioemotional functioning. Am. J. Intellect. Dev. Disabil. 115, 234–249. 10.1352/1944-7558-115.3.234 20441393

[B27] Ewles G. Clifford T. Minnes P. (2014). Predictors of advocacy in parents of children with autism spectrum disorders. J. Dev. Disabil. 20, 73–82.

[B28] Ferrer F. Vilaseca R. Guàrdia Olmos J. (2017). Positive perceptions and perceived control in families with children with intellectual disabilities: relationship to family quality of life. Qual. Quant. 51, 903–918. 10.1007/s11135-016-0318-1

[B29] Firth I. Dryer R. (2013). The predictors of distress in parents of children with autism spectrum disorder. J. Intellect. Dev. Disabil. 38, 1–9. 10.3109/13668250.2013.773964 23509963

[B30] García-López C. Sarriá E. Pozo P. (2016a). Parental self-efficacy and positive contributions regarding autism spectrum condition: an actor-partner interdependence model. J. Autism Dev. Disord. 46, 2385–2398. 10.1007/s10803-016-2771-z 27007725

[B31] García-López C. Sarriá E. Pozo P. (2016b). Multilevel approach to gender differences in adaptation in father-mother dyads parenting individuals with Autism Spectrum Disorder. Res. Autism Spectr. Disord. 28, 7–16. 10.1016/j.rasd.2016.04.003

[B32] García-López C. Sarriá E. Pozo P. Recio P. (2016c). Supportive dyadic coping and psychological adaptation in couples parenting children with autism spectrum disorder: the role of relationship satisfaction. J. Autism Dev. Disord. 46, 3434–3447. 10.1007/s10803-016-2883-5 27506645

[B33] García-Villamisar D. Polaino-Lorente A. (1992). Evaluación del autismo infantil: una revisión de los instrumentos escalares y observacionales. Act. Ped. Esp. 50, 383–388.

[B34] Gau S. S. Chou M. C. Chiang H. L. Lee J. C. Wong C. C. Chou W. J.. (2012). Parental adjustment, marital relationship, and family function in families of children with autism. Res. Autism Spectr. Disord. 6, 263–270. 10.1016/j.rasd.2011.05.00722512293

[B35] Goetz G. L. Rodriguez G. Hartley S. L. (2019). Actor-partner examination of daily parenting stress and couple interactions in the context of child autism. J. Fam. Psychol. 33, 554–564. 10.1037/fam0000527 30973257 PMC6897294

[B36] Griffith G. M. Hastings R. P. Nash S. Hill C. (2010). Using matched groups to explore child behavior problems and maternal well-being in children with Down syndrome and autism. J. Autism Dev. Disord. 40, 610–619. 10.1007/s10803-009-0906-1 19936904

[B37] Hastings R. P. (2003). Child behaviour problems and partner mental health as correlates of stress in mothers and fathers of children with autism. J. Intellect. Disabil. Res. 47, 231–237. 10.1046/j.1365-2788.2003.00485.x 12787155

[B38] Hastings R. P. Allen R. McDermott K. Still D. (2002). Factors related to positive perceptions in mothers of children with intellectual disabilities. J. Appl. Res. Intellect. Disabil. 15, 269–275. 10.1046/j.1468-3148.2002.00104.x18173571

[B39] Hastings R. P. Brown T. (2002). Behavioral problems of children with autism, parental self-efficacy, and mental health. Am. J. Ment. Retard. 107, 222–232. 10.1352/0895-8017(2002)107<0222:BPOCWA>2.0.CO;2 11966335

[B40] Hastings R. P. Kovshoff H. Ward N. J. Espinosa F. D. Brown T. Remington B. (2005). Systems analysis of stress and positive perceptions in mothers and fathers of pre-school children with autism. J. Autism Dev. Disord. 35, 635–644. 10.1007/s10803-005-0007-8 16177837

[B41] Hastings R. P. Taunt H. M. (2002). positive perceptions in families of children with developmental disabilities. Am. J. Ment. Retard. 107, 116–127. 10.1352/0895-8017(2002)107<0116:PPIFOC>2.0.CO;2 11853529

[B42] Jones L. Hastings R. P. Totsika V. Keane L. Rhule N. (2014). Child behavior problems and parental well-being in families of children with autism: The mediating role of mindfulness and acceptance. Am. J. Intellect. Dev. Disabil. 119, 171–185. 10.1352/1944-7558-119.2.171 24679352

[B43] Kakkar D. (2020). “Drift-diffusion model parameters underlying cognitive mechanism and perceptual learning in autism spectrum disorder,” in Soft Computing: Theories and Applications, eds Pant M. Sharma T. K. Verma O. P. Singla R. Sikander A. (Singapore: Springer) 847–857.

[B44] Karst J. S. Van Hecke A. V. (2012). Parent and family impact of autism spectrum disorders: a review and proposed model for intervention evaluation. Clin. Child Fam. Psychol. Rev. 15, 247–277. 10.1007/s10567-012-0119-6 22869324

[B45] Kayfitz A. D. Gragg M. N. Robert Orr R. (2010). Positive experiences of mothers and fathers of children with autism. J. Appl. Res. Intellect. Disabil. 23, 337–343. 10.1111/j.1468-3148.2009.00539.x

[B46] Kenny D. Kashy D. Cook W. (2006). Dyadic Data Analysis. New York, NY: The Guilford Press.

[B47] Kenny D. A. (1996). Models of non-independence in dyadic research. J. Soc. Pers. Relat. 13, 279–294. 10.1177/0265407596132007

[B48] Kenny D. A. Cook W. L. (1999). Partner effects in relationships research: conceptual issues, analytic difficulties, and illustrations. Pers. Relat. 6, 433–448. 10.1111/j.1475-6811.1999.tb00202.x

[B49] King G. Baxter D. Rosenbaum P. Zwaigenbaum L. Bates A. (2009). Belief systems of families of children with autism spectrum disorders or Down syndrome. Focus Autism Other Dev. Disabil. 24, 50–64. 10.1177/1088357608329173

[B50] Kline R. B. (2005). Principles and Practice of Structural Equation Modeling, 2nd Edn. New York, NY: The Guilford Press.

[B51] Konstantareas M. M. Homatidis S. (1989). Assessing child symptom severity and stress in parents of autistic children. J. Child Psychol. Psychiatry 30, 459–470. 10.1111/j.1469-7610.1989.tb00259.x 2745596

[B52] Konstantareas M. M. Papageorgiou V. (2006). Effects of temperament, symptom severity and level of functioning on maternal stress in Greek children and youth with ASD. Autism 10, 593–607. 10.1177/1362361306068511 17088275

[B53] Lee G. K. (2009). Parents of children with high functioning autism: how well do they cope and adjust? J. Dev. Phys. Disabil. 21, 93–114. 10.1007/s10882-008-9128-2

[B54] Lim K. K. Chong W. H. (2017). Moderating effect of child's autism spectrum disorder (ASD) diagnosis on benefit finding and negative affect of parents. Am. J. Orthopsychiatry 87, 357–364. 10.1037/ort0000251 28253014

[B55] Little L. (2002). Differences in stress and coping for mothers and fathers of children with Asperger's syndrome and nonverbal learning disorders. Pediatr. Nurs. 28, 565–570. 12593341

[B56] Lovell B. Wetherell M. A. (2020). Exploring the moderating role of benefit finding on the relationship between child problematic behaviors and psychological distress in caregivers of children with ASD. J. Autism Dev. Disord. 50, 617–624. 10.1007/s10803-019-04300-w 31724121

[B57] Manning M. M. Wainwright L. Bennett J. (2011). The double ABCX model of adaptation in racially diverse families with a school-age child with autism. J. Autism Dev. Disord. 41, 320–331. 10.1007/s10803-010-1056-1 20623169

[B58] McCubbin H. I. Patterson J. M. (1983). “The family stress process: The Double ABCX model of adjustment and adaptation”, in Social Stress and the Family: Advances and Developments in Family Stress Theory and Research, eds McCubbin H. I. Sussman M. B. Patterson J. M. (New York, NY: The Haworth Press) 7–37.

[B59] McGrew J. H. Keyes M. L. (2014). Caregiver stress during the first year after diagnosis of an autism spectrum disorder. Res. Autism Spectr. Disord. 8, 1373–1385. 10.1016/j.rasd.2014.07.011

[B60] Meleady J. Clyne C. Braham J. Carr A. (2020). Positive contributions among parents of children on the autism spectrum: a systematic review. Res. Autism Spectr. Disord. 78:101635. 10.1016/j.rasd.2020.10163522250194

[B61] Mullins J. B. (1987). Authentic voices from parents of exceptional children. Fam. Relat. 36, 30–33. 10.2307/584643

[B62] Nieto C. López B. Gandía H. (2017). Relationships between atypical sensory processing patterns, maladaptive behavior and maternal stress in Spanish children with autism spectrum disorder. J. Intellect. Disabil. Res. 61, 1140–1150. 10.1111/jir.12435 29154486

[B63] Nordahl-Hansen A. Hart L. Øien R. A. (2018). The scientific study of parents and caregivers of children with ASD: a flourishing field but still work to be done. J. Autism Dev. Disord. 48, 976–979. 10.1007/s10803-018-3526-9 29502150

[B64] Osborne L. A. McHugh L. Saunders J. Reed P. (2008). Parenting stress reduces the effectiveness of early teaching interventions for autistic spectrum disorders. J. Autism Dev. Disord. 38, 1092–1103. 10.1007/s10803-007-0497-7 18027079

[B65] Pakenham K. I. Sofronoff K. Samios C. (2004). Finding meaning in parenting a child with Asperger syndrome: correlates of sense making and benefit finding. Res. Dev. Disabil. 25, 245–264. 10.1016/j.ridd.2003.06.003 15134791

[B66] Patterson J. M. (1988). Families experiencing stress: I. The Family Adjustment and Adaptation Response Model: II. Applying the FAAR Model to health-related issues for intervention and research. Fam. Systems Med. 6, 202–237. 10.1037/h0089739

[B67] Paynter J. Riley E. Beamish W. Davies M. Milford T. (2013). The Double ABCX model of family adaptation in families of a child with an autism spectrum disorder attending an Australian early intervention service. Res. Autism Spectr. Disord. 7, 1183–1195. 10.1016/j.rasd.2013.07.006

[B68] Pozo P. Sarriá E. (2014). A global model of stress in mothers and fathers of individuals with autism spectrum disorders (ASD). Anal. Psicol. 30, 180–191. 10.6018/analesps.30.1.140722

[B69] Pozo P. Sarriá E. Brioso A. (2011). “Psychological adaptation in parents of children with autism spectrum disorders,” in A Comprehensive Book on Autism Spectrum Disorders, ed Mohammadi M. R. (Rietja: InTech) 107–130.

[B70] Reid M. Fesalbon M. Mendoza E. Alvord M. K. Rich B. A. (2019). Examining the relationship between parental symptomatology and treatment outcomes in children with autism spectrum disorder. J. Autism Dev. Disord. 49, 4681–4685. 10.1007/s10803-019-04151-5 31375972

[B71] Reitman D. Currier R. O. Stickle T. R. (2002). A critical evaluation of the Parenting Stress Index-Short Form (PSI-SF) in a head start population. J. Clin. Child Adolesc. Psychol. 31, 384–392. 10.1207/S15374424JCCP3103_10 12149976

[B72] Rodriguez G. Hartley S. L. Bolt D. (2019). Transactional relations between parenting stress and child autism symptoms and behavior problems. J. Autism Dev. Disord. 49, 1887–1898. 10.1007/s10803-018-3845-x 30623270 PMC6897296

[B73] Samios C. Pakenham K. I. Sofronoff K. (2009). The nature of benefit finding in parents of a child with Asperger syndrome. Res. Autism Spectr. Disord. 3, 358–374. 10.1016/j.rasd.2008.08.00315134791

[B74] Samios C. Pakenham K. I. Sofronoff K. (2012). Sense making and benefit finding in couples who have a child with Asperger syndrome: an application of the Actor-Partner Interdependence Model. Autism 16, 275–292. 10.1177/1362361311418691 21949006

[B75] Sarriá E. Pozo P. (2015). “Coping strategies and parents' positive perceptions of raising a child with autism spectrum disorders,” in: *Autism Spectrum Disorder-Recent Advance*, ed Fitzgerald M. (Rietja: InTech) 51–79. 10.5772/58966

[B76] Schlebusch L. Dada S. (2018). Positive and negative cognitive appraisal of the impact of children with autism spectrum disorder on the family. Res. Autism Spectr. Disord. 51, 86–93. 10.1016/j.rasd.2018.04.005

[B77] Schopler E. Reichler R. J. Renner B. R. (1988). The Childhood Autism Rating Scale (CARS). Western Psychological Services.

[B78] Seligman M. Darling R. B. (2007). Ordinary Families, Special Children, 3rd Edn. New York, NY: The Guildford Press.

[B79] Shalev R. A. Lavine C. Di Martino A. (2020). A systematic review of the role of parent characteristics in parent-mediated interventions for children with autism spectrum disorder. J. Dev. Phy. Disabil. 32, 1–21. 10.1007/s10882-018-9641-x

[B80] Stuart M. McGrew J. H. (2009). Caregiver burden after receiving a diagnosis of an autism spectrum disorder. Res. Autism Spectr. Disord. 3, 86–97. 10.1016/j.rasd.2008.04.006

[B81] Summers J. A. Behr S. K. Turnbull A. P. (1989). “Positive adaptation and coping strengths of families who have children with disabilities,” in Support for Caregiving Families: Enabling Positive Adaptation to Disabilities, eds Singer G. H. S. Irvin L. K. (Baltimore, MD: Brookes)27–40.

[B82] Suzuki K. Hiratani M. Mizukoshi N. Hayashi T. Inagaki M. (2018). Family resilience elements alleviate the relationship between maternal psychological distress and the severity of children's developmental disorders. Res. Dev. Disabil. 83, 91–98. 10.1016/j.ridd.2018.08.006 30145457

[B83] Taunt H. M. Hastings R. P. (2002). Positive impact of children with developmental disabilities on their families: a preliminary study. Educ. Train. Mental Retard. Dev. Disabil. 37, 410–420.

[B84] Tejero A. Guimerá E. M. Farré J. (1986). Uso clínico del HADS (Hospital Anxiety and Depression Scale) en población psiquiátrica: un estudio de su sensibilidad, fiabilidad y validez. Rev. Depart. Psiq. Facul. Med. Barc. 13, 233–238.

[B85] Turnbull A. P. Summers J. A. Brotherson M. J. (1986). “Family life cycle: theoretical and empirical implications and future directions for families with mentally retarded members,” in Families of Handicapped Persons: Research, Programs, and Policy Issues, eds Gallagher J. Vietze P. (Baltimore, MD: Brookes) 45–65.

[B86] Wadhera T. Kakkar D. (2019). Diagnostic assessment techniques and non-invasive biomarkers for Autism Spectrum Disorder. Int. J. E-Health Med. Commun. 10, 79–95. 10.4018/IJEHMC.201907010530473705

[B87] Wadhera T. Kakkar D. (2020). Interdisciplinary Approaches to Altering Neurodevelopmental Disorders. (AMDTC Book Series). Hershey, PA: IGI Global.

[B88] Waizbard-Bartov E. Yehonatan-Schori M. Golan O. (2019). Personal growth experiences of parents to children with autism spectrum disorder. J. Autism Dev. Disord. 49, 1330–1341. 10.1007/s10803-018-3784-6 30367347

[B89] Wong C. C. Mak W. W. Liao K. Y. H. (2016). Self-compassion: a potential buffer against affiliate stigma experienced by parents of children with autism spectrum disorders. Mindfulness 7, 1385–1395. 10.1007/S12671-016-0580-2

[B90] Xue J. Ooh J. Magiati I. (2014). Family functioning in Asian families raising children with autism spectrum disorders: the role of capabilities and positive meanings. J. Intellect. Disabil. Res. 58, 406–420. 10.1111/jir.12034 23510076

[B91] Yorke I. White P. Weston A. Rafla M. Charman T. Simonoff E. (2018). The association between emotional and behavioral problems in children with autism spectrum disorder and psychological distress in their parents: A systematic review and meta-analysis. J. Autism Dev. Disord. 48, 3393–3415. 10.1007/s10803-018-3605-y 29777471 PMC6153902

[B92] Zaidman-Zait A. Mirenda P. Duku E. Szatmari P. Georgiades S. Volden J.. (2014). Examination of bidirectional relationships between parent stress and two types of problem behaviour in children with autism spectrum disorder. J. Autism Dev. Disord. 44, 1908–1917. 10.1007/s10803-014-2064-3 24550079

[B93] Zaidman-Zait A. Mirenda P. Zumbo B. D. Georgiades S. Szatmari P. Bryson S.. (2011). Factor analysis of the Parenting Stress Index-Short Form with parents of young children with autism spectrum disorders. Autism Res. 4, 336–346. 10.1002/aur.213 21882359

[B94] Zaidman-Zait A. Mirenda P. Zumbo B. D. Wellington S. Dua V. Kalynchuk K. (2010). An item response theory analysis of the Parenting Stress Index-Short Form with parents of children with autism spectrum disorders. J. Child Psychol. Psychiatry 51, 1269–1277. 10.1111/j.1469-7610.2010.02266.x 20546082

[B95] Zigmond A. S. Snaith R. P. (1983). The Hospital Anxiety and Depression Scale (HADS). Acta Psychiatr. Scand. 67, 361–370. 10.1111/j.1600-0447.1983.tb09716.x 6880820

